# Genetic Diversity of Newcastle Disease Virus and Its Implications for Vaccine Development

**DOI:** 10.3390/vetsci12090858

**Published:** 2025-09-04

**Authors:** Olga A. Kondakova, Alexey A. Agranovsky, Ekaterina M. Ryabchevskaya, Elizaveta P. Umarova, Dmitriy L. Granovskiy, Stepan E. Toropov, Ekaterina A. Evtushenko, Nikolai A. Nikitin, Olga V. Karpova

**Affiliations:** Department of Virology, Faculty of Biology, Lomonosov Moscow State University, 119234 Moscow, Russia

**Keywords:** Newcastle disease virus, genotypes, vaccines

## Abstract

The Newcastle disease virus (NDV), an avian paramyxovirus, induces the highly contagious Newcastle disease in poultry. Newcastle disease outbreaks, common in many developing countries, have been recorded worldwide for a century. Poultry, even vaccinated stocks, together with wild and synanthropic birds, serve as reservoirs of NDV. Despite the extensive use of commercial NDV vaccines, Newcastle disease outbreaks frequently occur in vaccinated chickens, resulting in great economic losses. The primary limitation of commercial Newcastle disease vaccines is their restricted compatibility with emerging novel NDV strains. The advancement of vaccines and vaccination techniques is anticipated to reduce the propagation of pathogenic NDV strains and consequently alleviate losses in poultry production. This review examines the NDV genotypes and strains implicated in both current and historical Newcastle disease outbreaks, and evaluates existing and candidate NDV vaccines, emphasizing recent innovations and novel techniques. Our aim was to delineate critical subjects for future inquiry and to furnish extensive data that could aid researchers in understanding the current advancements and existing problems in Newcastle disease vaccination prophylactics. The emergence of a new generation of vaccines employing advanced technologies may substantially improve the efficacy of Newcastle disease prevention and control.

## Simple Summary

The Newcastle disease virus causes a highly contagious disease in poultry. Newcastle disease outbreaks, common in numerous developing countries, have been recorded worldwide for a century. Poultry, including vaccinated populations, along with wild birds, serve as reservoirs for the virus. The main limitation of commercial Newcastle disease vaccines is their limited compatibility with emerging virus strains. The development of new vaccines is expected to decrease the spread of pathogenic Newcastle disease virus strains and thereby mitigate losses in poultry production. This review analyzes the virus strains associated with both contemporary and historical Newcastle disease outbreaks, and evaluates current and candidate vaccines, highlighting recent advancements. The development of a new generation of vaccines is expected to significantly enhance the efficacy of Newcastle disease prevention and control.

## 1. Introduction

The Newcastle disease virus (NDV), an avian paramyxovirus, causes the highly contagious Newcastle disease in chickens, marked by increased mortality rates and substantial economic losses. Outbreaks of Newcastle disease, endemic in many developing countries, have been documented globally for a century. The World Organization for Animal Health (WOAH) categorizes highly pathogenic variants of Newcastle disease as a reportable disease. Poultry, even vaccinated individuals, together with wild and synanthropic birds, act as reservoirs of NDV [1]. The most efficient approach to managing Newcastle disease in countries with advanced commercial poultry industries is preventive vaccination. Notwithstanding the widespread administration of commercial vaccines, outbreaks of Newcastle disease are often documented in vaccinated chicken, leading to economic losses from mortality, stunted growth, and reduced egg production. The principal drawback of commercial Newcastle disease vaccines is their limited compatibility with the emerging novel NDV strains and genotypes [2,3,4,5,6,7]. The development of innovative vaccines and vaccination methods is expected to diminish the transmission of pathogenic NDV strains and, as a result, mitigate losses in chicken production.

This review examines existing and candidate NDV vaccines, emphasizing recent vaccine developments over the past five years and the implementation of innovative approaches. Our objective was to highlight key topics for future research and to present comprehensive data that could assist researchers in recognizing the current state of the art and prevailing challenges in Newcastle disease vaccine prophylactics.

## 2. Structure of NDV and Functions of Virus-Specific Proteins

**Figure 1:**
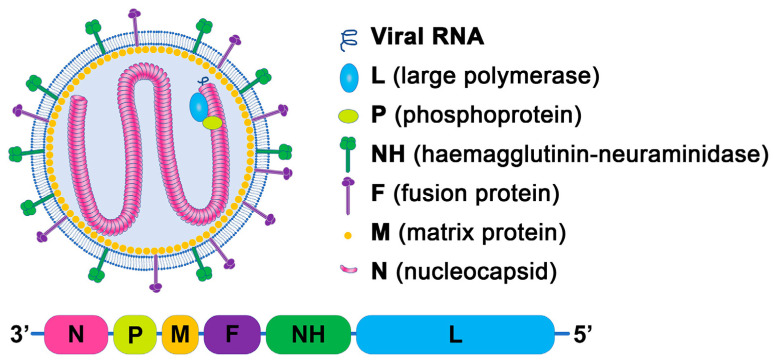
Schematic diagram of Newcastle disease virus structure and genome organization.

NDV has enveloped pleomorphic particles of 100–500 nm in diameter containing non-segmented negative-sense genomic RNA of about 15.000 nt. In 3′ to 5′ direction, the genome encompasses the genes for the nucleocapsid protein (N), phosphoprotein (P), matrix protein (M), fusion protein (F), haemagglutinin-neuraminidase (HN), and large replicase protein (L). The two supplementary non-structural proteins, V and W, function as antagonists of interferon alpha-beta, with their mRNAs generated through RNA editing during the transcription of the P gene [8,9]. N, P, and L interact with the viral RNA to produce the NDV nucleoprotein, which is involved in the transcription and replication of the virus RNA. Protein M facilitates particle maturation and budding during the late infection phase; in mature particles, M paves the virion membrane interior and interacts with the viral nucleoprotein and surface glycoproteins [10] (Figure 1).

The surface glycoproteins F and HN are critical determinants of tissue tropism and pathogenicity in NDV strains. Protein F facilitates the fusion of NDV with the host cell membrane. This protein is initially produced in an inactive state (F0), which is then cleaved by a host cell protease into the F1 and F2 fragments, retained together by disulfide links. The cleavage of F0 is essential for rendering NDV particles infectious. The NDV strains exhibiting low and high virulence vary in the amino acid sequences at the F0 proteinase cleavage sites. The F protein cleavage site of the virulent and avirulent NDV strains include the sequences 112R/K–R–Q/K-R/R–K/R–R–F117 and 112G/E–K/R–Q–G/E–R–L117, respectively [11]. HN is a type II integral membrane protein that performs essential roles in NDV infection and pathogenesis, including the activation of the F protein and binding to cell receptors containing sialic acid. Moreover, the proper maturation, budding, and egress of de novo produced NDV particles are contingent upon the HN activity, which modifies sialic acid residues [12,13,14,15,16,17].

## 3. NDV Taxonomy, Genetic Variability, and Epidemiology

NDV belongs to the *Orthoavulavirus* genus (subfamily *Avulavirinae*, family *Paramyxoviridae*); as per the International Committee for the Taxonomy of Viruses (ICTV), the virus is designated as *Orthoavulavirus javaense* [18,19]. Although all known NDV isolates belong to the same serotype, there exists significant genetic and antigenic heterogeneity across the viral strains [20]. Multiple investigations have demonstrated antigenic variations within strains of the same genotype and between phylogenetically distinct NDV genotypes [21,22,23]. The identification of restriction sites in the *F* gene provided the foundation for the current classification of NDV strains, facilitating the distinction of six genotypes [24]. The NDV isolates were categorized into two main classes, class I and class II, based on the length of their whole genomes [25]. Subsequently, phylogenetic analyses of the whole gene F sequences were employed to establish a unified nomenclature for viral isolates [26]. According to this nomenclature system, class I consists of a single genotype I (GI), whereas class II encompasses 15 genotypes, 10 of which were previously recognized (GII-GIX and GXI) and five of which are novel (GX, GXII-GXV). Diel et al. (2012) further categorized these genotypes into subgenotypes [26]. The subsequent studies have revealed three additional genotypes, designated GXVI, GXVII, and GXVIII [27,28]. A revised nomenclature and categorization scheme for NDV isolates was proposed in 2019 [29]. Class I includes genotype I, which is separated into three subgenotypes (1.1.1, 1.1.2, and 1.1.3). Class II consists of at least 20 genotypes, some of which are further categorized into subgenotypes [29].

Most class I isolates have minimal virulence in chickens and have been isolated from wild birds and shorebirds. In 1990, an epidemic of a highly virulent class I NDV strain in chickens was recorded in Ireland. Subsequent research indicates that this isolate may have originated from a low-virulence strain circulating among waterfowl, due to point mutations at the F protein cleavage site [1]. A recent epidemiological survey in China indicates that NDV class I strains may exhibit heightened virulence due to changes in the *F* gene, and the ongoing circulation of these strains in their natural hosts may lead to the emergence of more virulent strains [30].

Class II strains possess a wider pathogenicity range, are commonly isolated from poultry, and demonstrate increased genetic heterogeneity (Table 1). Table 1 also encompasses the distinct genotypes and subgenotypes identified post-2019, together with the strains categorized by Dimitrov and associates. In North-Eastern India, a 2023 epidemic of Newcastle disease among vaccinated chickens resulted in a mortality rate of 90–100%. A phylogenetic reconstruction utilizing complete *F* gene sequences indicates that the implicated NDV strain is a unique genotype proposed as GXXII [5] (Table 1).

The varying pathogenicity and symptomatology of NDV strains in chickens is a distinctive trait. The lack of pathognomonic signs impedes the capacity for accurate diagnoses. NDV strains are categorized into three pathotypes: lentogenic, which induce subclinical infections with mild respiratory or enteric symptoms; mesogenic, which result in respiratory infections with moderate mortality; and velogenic, which are highly virulent and lead to elevated mortality rates in susceptible chickens. Velogenic strains are classified into neurotropic, which impact the central nervous system, and viscerotropic, which largely affect the intestinal tract. Mesogenic and velogenic strains are classified as virulent strains responsible for Newcastle disease and must be registered in compliance with WOAH regulatory guidelines. The virulent strains must satisfy one of the following criteria, according to WOAH: contain several basic amino acid residues at the C-terminus and the Phe-117 in protein F, or contain an intracerebral pathogenicity score (ICPS) of 0.7 or above in day-old chickens. Low-virulence lentogenic isolates, commonly employed as live vaccines, are not subject to registration [11,31]. Table 1 presents the pathogenicity of various NDV genotypes obtained from wild and poultry species.

**Table 1:** Genetic diversity and global distribution of NDV strains.

Continent	Genotype	Subgenotype	Hosts	Pathogenicity for Chicken	References
Eurasia	I	I.1.1	Poultry, wild and migratory waterfowl	Low virulent	[32,33,34,35]
		I.2	Wild and domestic waterfowl		
		I.1.2.1	Wild and migratory waterfowl and landbirds		
	II	-	Gallinaceous poultry and domestic waterfowl, wild birds, peridomestic birds	Low virulent and virulent	[32,35,36,37]
	III	-	Chickens and domestic waterfowl	Virulent	[35,38]
	IV	-	Poultry	Virulent	[35,39]
	V	V.1, V.2	Poultry, wild birds	Virulent	[32,35,40]
	VI	VI.1.1, VI.2.1 VI.1.2.2.2 VI.2.1.1.2.1 VI.2.1.1.2.2.	Wild and domestic pigeons, poultry	Low virulent and virulent	[29,33,35,41,42,43]
	VII	VII.1.1	Poultry, domestic and migratory waterfowl, wild birds, peridomestic birds	Virulent	[29,34,35,36,40,42,44,45,46,47]
		VII.1.2	Chickens, pigeons, wild migratory birds		[29,35,36]
		VII.2	Chickens, wild birds		[4,7,37,44,48,49,50]
			Vaccinated chicken	100% mortality	[6]
	VIII	-	Vaccinated chinese game fowl	100% morbidity and mortality	[3]
	IX	-	Poultry, wild birds	Virulent	[51,52]
	XII	XII.1, XII.2	Chickens, domestic geese	Virulent	[35,53,54]
	XIII	XIII.1	Vaccinated chicken	80% mortality	[2]
		XIII.2 XIII.2.1 XIII.2.2	Chickens	Virulent	[37,55,56,57]
		XIII.2.3 (N.V.)	Chickens	Virulent	[58]
	XX	-	Chickens	Virulent	[59]
		-	Pigeons	80% morbidity and mortality	[60]
	XXI	XXI.1.1 XXI.1.2	Pigeons	Virulent	[36,37,61]
	XXII (N.V.)	XXII.1, XXII.2.	Vaccinated chicken	90–100% mortality	[5]
Africa	I	N.I.	Chickens, wild and domestic waterfowl	Low virulent	[62,63]
		I.1	Chickens		[64]
	II	-	Poultry and wild birds	Low virulent and virulent	[62,63,64]
	III	-	Chickens and domestic waterfowl	Virulent	[28]
	IV	-	Poultry	Virulent	[62]
	V	N.I.	Chickens	Virulent	[62]
		V.3 (N.V.)	Chickens	Virulent	[65]
	VI	VI.1.1	Pigeons and parrot	Low virulent and virulent	[62]
		VI.1.2	Chickens		
		VI.1.2.1.1	Pigeons and Chickens		
		VI.1.2.1.2	Pigeons and doves		
		VI.1.2.2.1	Chickens		
	VII	VII.1.1	Poultry, wild birds (migratory and non-migratory)	Virulent	[62,66,67,68,69]
		VII.2	Chickens		[62,70,71]
	VIII	-	Chickens	Virulent	[63,72]
	XI	-	Poultry	Virulent	[73]
	XIII	XIII.1. XIII.2	Poultry	Virulent	[62]
	XIV	XIV.1, XIV.2	Chickens, village weaver	Virulent	[62]
	XVII	-	Poultry	Virulent	[62,74]
	XVIII	XVIII.1 XVIII.2	Chickens, wild birds	Virulent	[62,74]
	XX	-	Poultry	Virulent	[62]
	XXI	XXI.1.1 XXI.2	Chickens, pigeons	Virulent	[74,75]
	N.V.		Chickens	Virulent	[76]
North America	I	I.1.2.1 I.2	Wild and migratory waterfowl and landbird, domestic waterfowl	Low virulent	[32,35]
	II	-	Poultry and domestic waterfowl, wild birds	Low virulent and virulent	[32,35,77]
	V	V; V.I V.2	Chickens, wild birds	Virulent	[77,78]
	VI	VI.1.2.2.1 VI.1.2.1.1.1 VI.2.1.1.1	Pigeons, doves, chickens and poultry	Low virulent and virulent	[35,77,79,80]
	X	-	Wild waterfowl, turkeys	Low virulent	[29,77]
	XVI	-	Chickens	Virulent	[27,77]
	XIX	-	Cormorant, pelicans, gulls, chickens	Virulent	[77]
South America	I	I.1.1	Chickens	Low virulent	[81]
	II	-	Chickens	Low virulent	[81]
	V	V.2	Chickens, wild birds	Virulent	[82,83]
	VI	VI.1.1 VI.1.2.1.2 VI.2.1.2 VI.2.1.1.1	Pigeons	Low virulent and virulent	[83,84]
	VII	VII.1.1	Chickens, fighting cock (*Gallus gallus*)	Virulent	[81,85,86]
	X	-	Wild waterfowl	Low virulent	[35]
	XII	XII.1	Chickens, peacock	Virulent	[81,87,88]
Australia	I	N.I.	Chickens	Virulent	[89]
	VI	VI. 2.1.1.2.2	Pigeons	Virulent	[90]

Abbreviation: N.V.—new variant, N.I.—No information.

## 4. Global Distribution and Panzootics of Newcastle Disease

The class II NDV genotypes have been implicated in the majority of Newcastle disease outbreaks and have contributed to at least five panzootics globally (Table 1 and Table 2) [1,29,44].

The NDV genotypes GII, GIII, and GIV were implicated in the first panzootic that took place from 1926 to 1960. The second panzootic occurring between 1960 and 1970 was attributed to the NDV GV. The genotype GVI initiated the third panzootic, starting with domestic pigeons in the late 1970s and later spreading to poultry and wild pigeons. The NDV GVII strains were implicated in the occurrence of the fourth and fifth panzootics, specifically subgenotypes GVII.1.1 and GVII.2. The GVII strains seem to be the main contributors to most Newcastle disease outbreaks across the three continents (Eurasia, Africa, South America) and represent the most significant risk to poultry production, even though GVI strains are found worldwide (Table 1).

Notwithstanding the extensive proliferation and persistent risk of Newcastle disease, several countries, including the United States, Canada, and Western European nations, have effectively instituted preventive strategies to mitigate the disease. Nonetheless, in some regions of Africa, South America, and Eurasia, Newcastle disease continues to threaten poultry production. It is important to note that wild birds serve as the natural reservoirs for NDV. Given that these wild species exhibit minimal to no signs of the disease and may transmit the virus across extensive distances, they facilitate the worldwide circulation of the virus throughout natural ecosystems [91]. Several factors hinder the comprehensive evaluation of NDV dissemination worldwide. Firstly, only cases of sickness attributed to virulent NDV strains in large scale chicken production are documented in most regions. Secondly, the extensive application of live NDV vaccinations may complicate the acquisition of accurate data regarding the virus’s distribution, as it becomes challenging to differentiate between vaccine and wild-type NDV strains using conventional diagnostic methods. Consequently, despite advancements in Newcastle disease control, the global scientific and veterinary societies persist in confronting challenges related to disease surveillance and prevention.

## 5. Current Vaccines for Newcastle Disease

Contemporary veterinary medicine predominantly employs live vaccinations utilizing low-virulent NDV strains to manage Newcastle disease. These include GII strains LaSota, B1, F, VG/GA, among others, as well as GI Ulster and V4. Due to their relative safety and the convenience of customizable mass administration, including watering, feeding, and aerosol treatments, these live vaccines have been predominantly employed. Despite the development of live vaccines utilizing virulent strains (e.g., Komorov and Mukteswar), their application was restricted owing to biosafety apprehensions [92,93]. In 2005, a velogenic GIII JS/7/05/Ch isolate was discovered, exhibiting 99.7% genomic identity with the vaccine strain Mukteswar, with some amino acid mutations in the HN gene that enhanced virulence [94,95]. The additional drawback of live vaccinations is the potential for immunization failure due to immunosuppression caused by co-infection or the presence of maternal antibodies in young chickens. Several live immunizations may induce respiratory problems and an associated risk of subsequent bacterial infections. The effectiveness of live vaccinations in specific immunization procedures is augmented by the injection of inactivated vaccines. Nonetheless, despite the rigorous immunization programs and diverse vaccine administration strategies, only a limited success in the disease control may be attained. Live vaccination cannot entirely inhibit virus shedding upon subsequent infection with either homologous or heterologous NDV strains, hence allowing for continued virus circulation in the environment [96,97,98,99]. This is especially dangerous in case of GVII isolates that may cause up to 100% mortality of vaccinated chickens [100]. Outbreaks of Newcastle disease are frequently reported in vaccinated poultry, potentially leading to significant economic losses.

Because viral RNA-dependent RNA polymerases have a high rate of mutations during RNA synthesis [101], NDV, like other RNA viruses, is evolving rapidly [102]. Hence, the primary factor contributing to the restricted efficacy of commercial NDV vaccines is the limited genetic and antigenic compatibility between the vaccine strains and those arising and circulating in nature. Reports of Newcastle disease outbreaks and concomitant increase in the number of NDV genotypes and subgenotypes in many countries (Table 1) suggests that total vaccination of poultry may provoke the virus escape from the immune response and the emergence of new virulent strains [29,35].

Recombinant virus vector vaccines have been under development for over 25 years, utilizing several platforms including fowlpox virus, turkey herpesvirus (HVT), and NDV. Currently, second-generation vector vaccines based on HVT expressing the NDV F protein are being implemented in veterinary practice [103]. However, the HVT platform is frequently employed in vaccine formulations for various other diseases, including avian influenza, avian bursal disease, avian infectious laryngotracheitis, and avian mycoplasmosis. The use of dual or multiple vaccines employing the same expression platform is expected to diminish the specific immune response. Vector vaccines are acknowledged for inducing a delayed immune reaction, occurring roughly four weeks after inoculation. Therefore, a booster vaccination with live vaccines is recommended to one-day-old chickens in regions where Newcastle disease is endemic. Third generation vector vaccines utilize the HVT platform to express two or more separate viral antigens [103]. The NDV itself is utilized as a vaccine platform. Experimental bivalent vaccines are currently under evaluation, utilizing low-virulence NDV as a platform for the expression of antigens from avian influenza virus, infectious bursal disease virus, avian metapneumovirus, infectious bronchitis virus, infectious laryngotracheitis virus and Marek’s disease virus [104,105,106,107,108,109,110]. Two bivalent vector vaccines targeting NDV and avian influenza virus subtype H5 have been implemented in veterinary practice in China and Mexico [103]. Still the maternal antibodies in chickens pose a significant problem in the practical use of vector vaccines. Experimental data indicate that preexisting antibodies against NDV and heterologous antigens may adversely affect vaccination efficacy [104,111]. Furthermore, there exists a technical challenge to maintain the cold chain during shipping and storage, as some vector vaccines strictly require shipping in liquid nitrogen.

## 6. Vaccines Under Development

### 6.1. Whole-Virion Vaccines

The development of novel whole-virion vaccines based on currently circulating NDV strains is a complex endeavor that necessitates balancing the preservation of essential antigenic features with vaccine safety. Various approaches are employed to achieve this goal, including the attenuation of the vaccine strain, the replacement of the F and HN genes in an avirulent vaccine strain with the corresponding genes from the strain of interest, and the inactivation of the virulent strain. Novel avirulent NDV strains are also under investigation as prospective vaccine candidates.

Several studies have successfully generated attenuated NDV strains through the reverse genetics of a virulent GVII strain, specifically by altering the cleavage site of protein F [112,113,114,115,116,117]. Despite advances with attenuated GVII specimens made over a decade ago, practical information on their use is lacking, and additional research is necessary to evaluate their safety [117]. A number of candidate chimeric vaccines have been created from low-virulent NDV strains, wherein the F and/or HN genes have been substituted with those from circulating highly virulent strains [118,119,120,121,122]. By replacing the HN gene with its equivalent from the highly pathogenic NDV GVII strain SG10, Bu et al. (2019) created the chimeric vaccine rLaSota-HN. Comparative testing showed that one-day-old chickens immunized with the commercial LaSota vaccine and rLaSota-HN vaccine had 90% and 100% protection against the homologous challenge, respectively. Even though rLaSota-HN only offered a minor protective advantage, it completely eliminated viral shedding in contrast to the LaSota-vaccinated cohort’s 50% reduction in shedding [118].

Dewidar and colleagues (2022) examined the protective efficacy of live and inactivated conventional vaccine GII LaSota and recombinant GVII vaccines represented by GII LaSota or GII VG/GA backbones with substitutions of the intrinsic F and HN genes with the genes from a virulent subgenotype VII.1.1. All vaccinated chickens had high levels of protection against the NDV GVII. GII LaSota provided 93.3% protection. Recombinant specimens provided protection ranging from 80% to 86.6%for LaSota-based GVII and from 93.3% to 100% for VG/GA-based GVII, contingent upon the vaccination protocol (using only live vaccine or combination of live and inactivated vaccine variants) employed. However, in the recombinant GVII-vaccinated cohort of chickens, viral shedding was markedly reduced in comparison to the GII LaSota group [96]. It should be noted that testing for NDV shedding has emerged as a critical criterion for assessing vaccine efficacy and has been incorporated as a mandated standard in China. In accordance with the established protocol, cloacal swabs from at least 7 out of 10 vaccinated chickens must have negative results for viral isolation on the fifth day post-challenge [123].

Recently, the predominant subgenotype GVII.2 strain was isolated from sick chickens in Bangladesh and utilized to manufacture an inactivated vaccine [124,125]. The vaccine’s clinical protection against the homologous virus strain following two consecutive injections with the Montanide ISA 70 adjuvant was around 100%. This outcome differed from the mere 57% protection against GVII.2 seen in chickens immunized with the commercial live vaccine GII LaSota [124,125]. Another study conducted in Bangladesh evaluated the effectiveness of enhanced vaccination against the local virulent strain GXIII.2 BD-C161/2010 using three distinct vaccines (live GII LaSota, inactivated GII LaSota, and inactivated GXIII.2 vaccine) in chickens previously primed with the live GII LaSota vaccine [98]. While all three boosters safeguarded the inoculated avians from mortality following the GXIII.2 challenge, the inactivated LaSota shown superiority over the live LaSota, and the GXIII.2 vaccine provided comprehensive clinical protection along with a marked decrease in viral shedding. This trial utilized inactivated vaccinations combined with incomplete Freund’s adjuvant [98].

Due to the relatively poor immunogenicity of inactivated vaccines, the selection of an appropriate adjuvant is crucial for augmenting the immune response. Kumar et al. (2023) developed an inactivated vaccine targeting the virulent Indian strain GVII and encapsulated the formulation in poly-(lactic-co-glycolic) acid (PLGA) polymeric nanoparticles, which acted as an adjuvant, protected the antigen from nonspecific degradation, and facilitated its delivery to antigen-presenting cells. Immunization of chickens with the candidate vaccine yielded 100% clinical protection against the GVII challenge, in contrast to the 80% protection provided by the commercial GII LaSota vaccine using a water-in-oil emulsion adjuvant [126]. The nano-NDV vaccination elicited a markedly elevated titre of specific IgY and titre of HI antibodies as well as cell-mediated immunity, demonstrating enhanced production of IFN-γ in comparison to the commercial oil-adjuvanted LaSota. This study did not assess the virus shedding [126].

### 6.2. Recombinant Virus Vector Vaccines

#### 6.2.1. Herpesvirus Vector Vaccines

In commercial HVT-based vector vaccines Vectormune^®^ ND and Innovax^®^ ND, the F proteins of NDV GI and GII are utilized as antigens. Currently, heterologous prime-booster immunization protocols with HVT vaccines are under evaluation and practical application. It has been observed that initial vaccination with Vectormune^®^ ND or Innovax^®^ ND, followed by a booster with inactivated or live LaSota vaccine, significantly reduces shedding of the heterologous NDV GVII. In these studies, the inactivated LaSota booster demonstrated superiority over the live LaSota regarding antigenic response [127].

In a different approach, the F protein of the presently circulating virulent NDV strains has been utilized as an antigen in HVT vectors. Jia et al. (2022) implemented HDR-CRISPR/Cas9 technology to generate an HVT vector containing the NDV GVII *F* gene. A 70% protection rate for one-day-old chickens against the homologous GVII virus was attained five weeks post-vaccination [128]. Calderón et al. (2022) employed the CRISPR/Cas9-mediated NHEJ repair pathway and Cre/LoxP recombination to create a recombinant HVT incorporating the *F* gene of GVII prevalent in Peru. The resultant vaccine conferred 100% protection against the homologous virus from 14 days post-vaccination and completely eliminated viral shedding on the day five post-challenge in vaccinated chickens [129].

Recently, Shi et al. (2024) used CRISPR/Cas9 for engineering two expression vectors by integrating the NDV GVII *F* gene into distinct loci of the HVT vector. A singular administration of each vaccine to one-day-old chickens yielded 100% protection against the homologous challenge for a minimum of 52 weeks post-vaccination, accompanied by a complete cessation of NDV shedding from days 3 to 10 post-vaccination [130].

#### 6.2.2. Adenovirus Vector Vaccines

Recombinant human adenoviruses have been suggested as potential multicistronic vectors for NDV surface glycoproteins, serving as an alternative to the previously mentioned vaccine vectors. Adenovirus vectors offer significant benefits, including a high level of safety, genetic stability, and the absence of preexisting human adenoviral immunity in avian species [131,132,133]. Researchers from China, the UK, the UAE, and Sudan have created a candidate vaccine employing recombinant adenovirus serotype 5 (rAd5) that integrates the HN gene of a virulent GVII strain C22 as the antigen, accompanied by chicken granulocyte monocyte colony-stimulating factor (ChGM-CSF) as the adjuvant [132]. Intramuscular administration of the rAd5-ChGM-CSF-HN vector in chickens resulted in superior HI antibody titers compared to immunizations with rAd5-HN (lacking the adjuvant gene) and the commercial live LaSota vaccine. Although all three vaccinated chicken groups exhibited 100% survival following the C22 strain challenge, the cohort administered rAd5-ChGM-CSF-HN demonstrated the lowest viral tissue burdens and decreased viral shedding [132]. Employing a comparable methodology, Xu et al. (2023) produced a recombinant adenovirus rAd5 that expresses the protein F of the virulent GVII strain DHN3. Post intramuscular inoculation, the chickens exhibited a 100% survival rate following the homologous DHN3 challenge, with 86% demonstrating no viral shedding [133].

A significant issue related to adenovirus vaccines and other vector vaccines is the imperative to rigorously maintain the cold chain during shipping and storage. This challenge has been addressed in recent studies concerning the adenovirus vector containing the *F* gene of NDV GVI circulating in Ethiopia [134,135]. The authors devised a cost-effective method for producing recombinant adenovirus in suspended HEK293 cells and developed optimized liquid and lyophilized formulations, both of which exhibited stability after six months of storage at +4 °C. A double intranasal immunization of 1–2-week-old chickens with the liquid adenovirus vaccine provided complete protection against the homologous NDV challenge on day 56 post-initial vaccination [134,135]. Virus shedding was not assessed in this work.

### 6.3. Plasmid DNA Vaccines

DNA vaccines have received heightened interest owing to their scalability, stability at ambient temperatures, and capacity to provoke a humoral and wider cellular response [136]. Nevertheless, DNA vaccines have certain difficulties in large-scale implementation, including limited in vivo cell transfection efficiency and comparatively low immunogenicity, which requires the recurrent administration of elevated vaccination dosages. The issues of DNA vaccine administration and suitable adjuvants have also been the focus of research [136].

Gao et al. (2020) devised a novel delivery strategy for a DNA plasmid encoding the HN protein of a highly pathogenic NDV GIX strain via a biodegradable PLGA-PEG-PLGA gel. The hydrogel-delivered DNA vaccine elicited both humoral and cellular immune responses in chickens, achieving 100% protection against the homologous virus challenge [137].

Xie et al. (2020) developed a DNA vaccine for NDV by integrating the *F* gene from a virulent NDV GVII.1.1 strain into the pCAGGS vector. The plasmid pCAG-IL-12, which expresses an auxiliary adjuvant, included the insert of the chicken interleukin-12 gene [138]. The pCAG-F DNA vaccine was administrated to chickens, either independently or in conjunction with pCAG-IL-12. In a group receiving dual inoculation with pCAG-F and pCAG-IL-12, the levels of neutralizing antibodies against F protein and concanavalin-A-stimulated lymphocyte proliferation were significantly elevated compared to a cohort inoculated exclusively with pCAG-F. The optimal defense against the homologous NDV challenge, diminished viral load, and reduced virus shedding were also noted in the doubly inoculated group. Electroporation demonstrated more efficacy than intramuscular injection for the delivery DNA vectors [138].

In 2024, two DNA vaccines targeting NDV were developed using the repeated cloning of the F and HN genes from two virulent GVII isolates obtained from Tanzania, utilizing a cytomegalovirus expression vector [139]. The double-gene plasmids contained the F and HN cassettes, whereas the triple-gene plasmids also included the gene for interferon λ3 (IFNλ3; IL-28b), which has shown a notable ability to augment specific adaptive immune responses, reduce inflammatory cytokine levels, and promote protective responses. Intramuscular administration of a triple-gene plasmid in chickens resulted in elevated HI titers of F- and HN-specific antibodies and conferred 80% protection against the GVII.2 challenge, surpassing the 60% protection offered by control inoculations with double-gene plasmids and the live LaSota vaccine [139]. These observations offer a compelling justification for employing IL-12 and IL-28b as adjuvants and enhancers of DNA vaccines against NDV.

It is important to acknowledge that these encouraging results were achieved using manual vaccination procedures, which are impractical for large-scale chicken production; hence, the advancement of scalable immunization techniques for DNA vaccines remains a subject for future investigation.

### 6.4. Live Bacterial Vaccine Vectors

Live bacterial vectors represent a promising platform for the expression of a gene of interest in homeothermic hosts following oral administration. *Mycobacterium bovis* (BCG) has predominantly been employed as a live vaccine vector against several bacterial and viral pathogens (reviewed in [140]). There are further instances of producing bacterial vectors that specifically target NDV strains. Thus, Ju et al. (2021) developed a recombinant strain of *Lactobacillus casei* with the plasmid coding for a fragment of the HN protein from a GVII NDV strain. Peroral administration of the candidate vaccine strain partially protected chickens from the virulent NDV strain challenge (80%) and had positive impacts on the performance, immunological function, gut development, and microbiota of vaccinated chickens [141]. Gao et al. (2019) generated a *Salmonella typhimurium* vector containing a plasmid that expresses a portion of the NDV GVII *F* gene and chicken interleukin-18 (chIL-18) as an adjuvant. The *S. typhimurium* strain χ11246, with regulated delayed lysis, was utilized to ensure vaccination safety [142]. Oral immunization with recombinant *S. typhimurium* elicited NDV-specific IgG antibodies in immunized chickens. The presence of chIL-18 markedly enhanced lymphocyte proliferation and elevated T cell production in vaccinated chickens. The expression of the F antigen with chIL-18 conferred 80% protection against the homologous viral challenge compared to only 60% protection in a cohort expressing the antigen without chIL-18. This research further underscores the potential of cytokines as molecular adjuvants to augment the immune response [142].

### 6.5. Recombinant Subunit Vaccines

Subunit vaccines composed of viral proteins or fragments thereof derived from diverse expression systems may serve as an alternative strategy to manage Newcastle disease (reviewed in [93,143]). The technologies for the design and isolation of biopharmaceutical recombinant antigens are recognized for their safety, scalability, and efficacy. The appropriate selection of an expression system is crucial, contingent upon antigen structure, size, manufacturing costs, and additional criteria (reviewed in [144]). Commercially available subunit NDV vaccines are presently unavailable.

#### 6.5.1. Plant-Based Expression Systems

Plants have advantages for protein expression, including cost efficiency, scalability, the capacity for eukaryotic-type post-translational modifications for eukaryotic viral proteins, and the lack of animal infections, hence ensuring the safety of plant-derived proteins. Recombinant protein in plants can be produced by two methods: stable transformation, which integrates the gene of choice into the nuclear or plastid genome, and transient expression, where the gene is temporarily introduced into plant cells. The procurement of stable transgenic plants is a complex, time-consuming, and labor-intensive process, accompanied by challenges with regulatory approval. Moreover, protein outputs from nuclear transformation are often quite low [144].

In 2006, Dow Agro Sciences LLC (Indianapolis, IN, USA) created a technique for extracting recombinant NDV HN protein from a suspension-cultured tobacco cell line, which became the first USDA-approved veterinary vaccine derived from plants. The vaccine provided more than 90% protection in chickens against lethal dose of NDV challenge. Notwithstanding the success in development and production, the company announced a decision not to commercialize the vaccine [145]. Ma et al. (2020) developed a subunit vaccine based on F protein expressed in stably transformed transgenic rice seeds. Complete protection against homologous strain (genotype V) was seen in chickens after twice intramuscular immunizations with the NDV F protein [146].

There are a number of studies addressed to the employing plant expression system to develop edible NDV vaccine. Guerrero-Andrade et al. (2006) demonstrated that the oral administration of ground kernels from transgenic maize expressing the NDV LaSota F protein resulted in the generation of specific antibodies, achieving complete protection of chickens against the virulent GV Chimalhuacan strain, comparable to the protection conferred by the attenuated LaSota vaccine [147].

Boroujeni et al. (2022) investigated the synthesis and immunogenicity of recombinant NDV proteins in transgenic tobacco plants. The expression levels of the HN and F proteins driven by the NtREL1 promoter in transgenic tobacco roots were quantified at 0.75% and 0.54% of the total soluble protein, respectively. The oral administration of transgenic tobacco hairy roots produced elevated levels of specific IgG and IgA antibodies in laboratory mice [148]. Motamedi et al. (2020) obtained transgenic rapeseed plants expressing HN-F fusion protein at rates of 0.18% of the total soluble protein; the chickens fed with the transgenic rapeseed extracts developed high levels of the specific and HI antibodies. The protection against NDV was not assessed in these studies [149].

An alternative approach for generating NDV subunit vaccines, which may enable rapid and efficient vaccine production, is transient expression in plants [150]. Efforts to express the full-size HN protein of a virulent NDV strain utilizing the pEAQ-HT vector in tobacco were not successful, as agroinfiltration resulted in tissue necrosis and reduced HN yields, probably due to illegitimate glycosylation of the HN in plants, leading to its degradation [151]. The pBI121 vector was used to clone a synthetic gene that had three tandem repeats of the NDV F protein epitope ^65^LLPNMPKDKEACAKAPL^81^ and four tandem repeats of HN protein epitope ^346^DEQDYQIR^353^. This gene was then used to temporarily express the chimeric polypeptide in maize leaves. However, even though mRNA and chimeric protein were effectively accumulated, the immune reaction of orally immunized rabbits was only moderate [152]. It should be noted that the limited success of these trials does not cast doubt on the concept of temporary expression systems; rather, it emphasizes the need for more extensive research with a range of expression vectors, NDV antigens, and plant species.

#### 6.5.2. Bacterial Expression Systems

Since its inception over 40 years ago for protein expression, *Escherichia coli* has consistently been the most prevalent and extensively studied prokaryotic expression system. *E. coli* expression systems offer a safe and cost-effective alternative to expression systems utilizing plant, yeast, mammalian, and insect cells. A number of works describe the development of *E. coli* systems for the expression and purification of NDV surface glycoproteins [153,154,155].

Lee et al. (2010) extracted the full-size HN (rHN) protein from an *E. coli* expression system and utilized the purified protein in immunization and challenge protection experiments. The incorporation of rHN into conventional inactivated Newcastle disease vaccines markedly enhanced the serum HI antibody titer in vaccinated chickens, whereas vaccination with rHN alone did not yield a significant increase. Significantly, the inclusion of rHN into the bivalent and multivalent ND+IC (ND+infectious coryza) and ND+IC+FC (ND+IC+fowl cholera) vaccines elevated the protection rate of immunized chickens from approximately 80–90% to 100% following exposure to a NDV velogenic strain (Sato, genotype III) [154].

Shahid et al. (2020) expressed the HN with a hexahistidine tag in *E. coli*, purified the protein via Ni-NTA affinity chromatography, and utilized it for immunization, resulting in the production of specific IgY antibodies in chickens, which was further enhanced in rHN formulations containing incomplete and complete Freund’s adjuvants [155].

Motamedi et al. (2014) employed in silico analysis to identify B- and T-cell epitopes within the HN and F proteins, facilitating the selection of potential epitopes in the HN head domain (aa 243–481), segments of the transmembrane and stem domains (aa 25–100), and the F protein region (aa 1 to 269) [156]. This group subsequently developed an *E. coli* expression method that enabled high yield levels of rHN and rF, achieving up to 40% of each viral protein relative to the total soluble bacterial protein. Both rHN and rF facilitated the effective generation of specific antibodies following parenteral immunization of mice; moreover antibodies produced after immunization with the commercial B1 NDV vaccine clearly recognized rHN and rF in ELISA assay [157].

This approach has been further utilized in the development of a polyepitopic vaccine aimed at controlling various NDV strains [158,159]. Immunogenic fragments, which include B- and T-cell epitopes, were selected in silico for HN (amino acids 296–366) and F (amino acids 42–182). A recombinant chimeric antigen comprising the F epitope, two tandem repeats of the HN epitope, and the heat-labile enterotoxin B subunit of *E. coli* (LTB adjuvant) was expressed in high yields in *E. coli*, purified, and utilized for the immunization of mice. Significant levels of specific IgG antibodies were observed in the sera of immunized mice (with complete and incomplete Freund’s adjuvants), comparable to those following immunizations with the commercial NDV B1. The antigen exhibited significant reactivity with sera from mice immunized with the B1 vaccine [159]. The incorporation of LTB and tandem repeats of the HN epitope in the chimeric antigen to elicit a heightened immune response is supported by the findings of Rahmani et al. (2023) [160]. Collectively, these findings suggest the potential for further investigation into the protective capacity of bacterially expressed NDV glycoprotein antigens. Currently, a wide range of polyepitopic vaccines targeting various genetically diverse viruses, utilizing conserved protein sequences, is under development [144,161,162,163].

### 6.6. Synthetic Peptide Vaccines

A multiepitopic peptide vaccine has been developed by a research groupcomposed of Pakistani, Chinese and French scientists [164]. Initially, promising epitopes of the NDV glycoproteins were identified using bioinformatic methods, followed by experimental assessments of their allergenicity, toxicity, and specific immunogenicity. The final peptide vaccine comprised 40 amino acids, incorporating the F protein B-cell epitope, the F cytotoxic T-lymphocyte (CTL) epitope, and the F and HN helper T-lymphocyte epitopes. The peptide demonstrated significant affinity and binding stability to chicken MHC1 and TLR4 receptors, as assessed through molecular docking and molecular dynamics simulations. Experimental evaluations demonstrated a significant immune response in both mice and chickens, with the vaccine inducing robust antibody production, as indicated by increasing HI antibody titers comparable to those in cohorts immunized with the mesogenic NDV strain Mukteswar (genotype III) [164].

### 6.7. Vaccines Based on Virus-like Particles

A promising approach to developing NDV vaccines involves the production of virus-like particles (VLP) composed of viral structural proteins. VLPs are expected to possess a structure and antigenic characteristics akin to those of the infectious virus, while the absence of viral RNA genome ensures antigen safety. The M protein is vital for VLP formation and budding, with M-HN and M-N interactions being necessary for the incorporation of HN and N proteins into VLPs. In contrast, the incorporation of the F protein necessitates interactions with both N and HN proteins [165]. NDV VLPs have been effectively generated in avian [165,166], insect [167,168,169], and plant cells [170]. McGinnes et al. (2010) produced the VLPs by the co-expression of M, N, F, and HN proteins of the NDV AV strain in a chicken embryo cell line. The VLP immunization of mice elicited a robust immune response, with IgG and HI antibody titers comparable to those observed in control immunizations using the inactivated NDV B1 strain. Furthermore, the VLP immunization elicited elevated T-cell response levels in comparison to the vaccine strain [166]. The co-expression of M, HN, and F proteins from the NDV GVII strain in insect cells led to the assembly of VLPs that surpassed the commercial LaSota vaccine regarding protection levels, virus tissue loading, and virus shedding in immunized chickens after homologous virus challenge [168]. In another study utilizing an insect cell expression system, virus-like particles (VLPs) composed of the M, HN, and F proteins of NDV GVII strain were generated and employed for immunization. Triple VLP immunizations in chickens conferred enhanced protection compared to double VLP inoculation with a subsequent B1 vaccine booster, despite the boosted group exhibiting higher HI levels [169].

VLPs incorporating the F and HN proteins of the virulent NDV GVII.2 strain were generated through transient co-expression assays in *Nicotiana benthamiana* plants [170]. The successful production of VLPs induced solely by F and HN is paradoxical, given that the NDV matrix protein is a prerequisite for VLP formation in all other tested expression systems [165,166,167,168,169]. This phenomenon may be due to specific intrinsic characteristics of the plant cells. Indeed, co-expression of M with HN and F did not increase the yields of VLPs in *N. bethamiana* [170]. Partially purified VLPs derived from plants were employed for the immunization of chickens, resulting in increased ELISA and HI titers of specific antibodies targeting the homologous GVII strain, as well as their cross-neutralizing activity against a GXIV strain. The authors calculated that one kilogram of infiltrated leaf material could effectively prime-boost vaccinate 10,000 chickens, demonstrating the cost-effectiveness and scalability of VLPs as a vaccine platform [170].

A bivalent candidate vaccine was created utilizing VLPs made from the M1 and HA proteins of avian influenza virus (AIV), in conjunction with a fusion protein with the cytoplasmic and transmembrane domains of AIV neuraminidase (NA) and the ectodomain of the HN protein from the NDV LaSota strain [171]. VLPs were purified from insect cells for use in chicken immunization, resulting in the generation of elevated levels of antibodies specific to IAV and NDV. The specific antibody and HI titers were similar to those elicited by commercial IAV and NDV vaccines, and the vaccinated chickens demonstrated complete protection against the NDV (strain F48E9, genotype IX) and AIV challenge [171]. A bivalent vaccine candidate was created utilizing the S1 protein of infectious bronchitis coronavirus (IBV) and the ectodomain of NDV F protein, which were individually fused to the TM and C-terminal domains of the IBV S protein [172]. After expression in insect cells, the chimeric VLPs were isolated and utilized for chicken immunization, resulting in high titers of specific antibodies against IBV and NDV, as well as increased levels of IL-4 and IFN-γ T cell cytokines. The VLP immunization provided complete protection against both viral pathogens [172].

Information on recent studies of candidate vaccines against Newcastle disease is summarized in Table 3.

## 7. Conclusions

Newcastle disease remains a considerable threat to both domestic and industrial poultry production worldwide, despite the widespread use of vaccines and the implementation of safety and quarantine measures. The primary cause of this phenomenon is the ongoing circulation of NDV among wild and domestic avian populations, which is associated with an increase in viral genetic diversity and the emergence of new virulent strains (Table 1). In RNA viruses, two primary mechanisms that contribute to genome plasticity are recognized: RNA recombination via the copy-choice mechanism and point mutations introduced by viral RNA polymerase [101,179]. Negative-sense RNA viruses employ ribonucleoproteins instead of naked RNA as templates for RNA replication [180]. This specific trait is expected to impede the ability of RNA polymerase to switch between templates and promote RNA recombination. Point mutagenesis, specifically in the genes encoding viral surface glycoproteins, still remains a critical mechanism in the evolution of NDV and other negative-sense viruses, enabling the ongoing emergence of new variants that can evade existing vaccines and population immunity.

Table 1 illustrates the genetic variability and the newly identified virulent isolates of NDV. The current panzootic in Eurasia, South America and Africa caused by NDV genotype VII variants, along with occasional NDV outbreaks worldwide, presents considerable concerns. NDV poses an issue to poultry production not only in endemic regions but also in countries with efficient control measures for Newcastle disease, such as the USA and Canada. This is partially explained by the circulation of avirulent viral strains in commercial poultry, which may evolve into virulent strains. Moreover, there exists a persistent risk of NDV dissemination by migratory wild birds across distant geographic areas, potentially instigating disease outbreaks even in nations with efficient Newcastle disease control and surveillance measures. The comprehensive evaluation of diverse genotypes and strains of NDV is impeded due to the recording of outbreaks primarily in commercial poultry, coupled with the extensive application of live vaccines that complicate NDV diagnostics and obscure the understanding of the virus prevalence and dissemination.

Hence, it is essential to enhance programs for managing Newcastle disease, particularly through the advancement of novel vaccines and the optimization of vaccination regimens, which would bolster poultry resistance to virulent NDV strains and mitigate outbreak-related losses.

Live and inactivated vaccines for NDV have been employed in poultry farms for more than sixty years. Most commercial vaccines utilize low-virulent strains from genotypes I and II as antigens, whereas the dominant velogenic strains are classified under genotypes III-IX and XI-XXII (Table 1). These vaccines mitigate clinical symptoms and reduce chicken mortality, yet do not prevent virus shedding and transmission. Instances of NDV outbreaks with high mortality in vaccinated chickens have also been documented (Table 1). Consequently, the inadequate efficacy of vaccination against current NDV threats is attributed to the disparities between the vaccine strains and the circulating strains, leading to diminished cross-protectivity against the latter. To address this issue, whole-virion vaccines derived from the prevalent virulent strains are under development (Table 3). The safety concerns of the development and administration of such vaccines have remained a subject of discussion.

Recombinant viral vector-based NDV vaccines are acknowledged for their considerable potential, despite certain challenges that necessitate further investigation. The primary issue concerning vector vaccines is the maintenance of the cold chain during transport and storage, which requires advancements in thermostabilization techniques. Another drawback is the necessity for individual immunization of chickens and the potential influence of maternal antibodies on vaccine efficacy. To improve immunization effectiveness, bivalent and multivalent vaccines employing NDV vectors that express antigens from multiple pathogens have been proposed. However, despite the clear advantages, such vaccines are expected to demonstrate diminished efficacy against NDV compared to live NDV vaccines.

Diverse alternative approaches have been proposed for the advancement of third-generation NDV vaccines, including recombinant subunit vaccines, VLPs, DNA vaccines, viral and live bacterial vector vaccines, and synthetic peptides (Table 3). The whole NDV F and HN proteins of circulating virulent strains, or their segments, function as the primary antigens in these vaccines. Encouraging data regarding the effective and targeted immune response were acquired for particular formulations. Particular emphasis is warranted in efforts to develop innovative adjuvants to improve vaccine efficacy. Nonetheless, the acquired data unequivocally necessitate additional thorough investigation and analysis. Particular attention must be directed towards the formulation of optimal immunization strategies utilizing various adjuvants and combinations of heterologous primary and booster vaccines. Several research studies are evaluating potential vaccines against commercially existing live and inactivated vaccines, focusing on both protection against NDV challenge and post-challenge viral shedding in inoculated chicken. These innovative approaches should be taken into consideration and evaluated in order to bring alternatives to conventional vaccines to market, addressing the challenges of protecting animals against Newcastle disease.

There is a clear need to develop broad-spectrum vaccines and strategies for rapid response to evolving NDV strains. Recombinant vaccines may serve as an effective tool due to their cost-effectiveness and adaptability to emerging virus variants. Achieving an effective vaccine may necessitate a multifaceted approach, incorporating heterologous immunization regimens. A promising strategy for developing broad-spectrum vaccines against various circulating NDV strains involves the utilization of multiepitope antigens paired with optimal adjuvants. The vaccines containing protective epitopes are anticipated to elicit an immune response targeting the immunodominant conservative glycoprotein regions of NDV strains prevalent in a specific area. This approach seems to offer benefits over commercial live and inactivated vaccines. A number of studies in this area have been initiated, and preliminary results have been reported.

Further complex research focused on optimizing vaccine strategies for the Newcastle disease management is critical. The introduction of a new generation of vaccines utilizing innovative technologies has the potential to significantly enhance the effectiveness of the disease prevention and control.

## Figures and Tables

**Table 2:** Newcastle disease panzootics described since 1926.

Panzootic	NDV Genotype (Subgenotype)	Years
1	GII, GIII, GIV	1926–1960
2	GV	1960–1970
3	GVI	1978–present
4	GVII (GVII.1.1)	1985–present
5	GVII (GVII.2).	2009–present

**Table 3:** Recent research in Newcastle disease candidate vaccines (2019–2025).

Antigen(s)	Approach/Expression System	Adjuvant	Delivery Route	Developers	Development Stage	References
Live vaccine						
NDV structural proteins	Reverse Genetics-Based attenuated strain	No	OC	FARVET S.A.C., Peru; University of Maryland, USA	Immunogenicity and protective efficacy in chicken	[119]
NDV structural proteins	Reverse Genetics-Based attenuated strain	No	ON	University Putra Malaysia, Malaysia	Immunogenicity and protective efficacy in chicken	[116]
NDV structural proteins	Chimeric vaccine, HN gene alteration in an existing vaccine strain	No	ON	China Agricultural University, China	HI serum titers and protective effectiveness in chicken	[118]
NDV structural proteins	Chimeric vaccine, modification of an existing attenuated strain	No	IO	US National Poultry Research Center, USA	HI serum titers and protective effectiveness in chicken	[173]
NDV structural proteins	Chimeric vaccine, HN and *F* genes alteration in an existing vaccine strain	No	OC	Beni-Suef University and Animal Health Research Institute, Egypt	HI serum titers and protective effectiveness in chicken	[96]
NDV structural proteins	Chimeric vaccine, *F* gene alteration in an existing virulent strain and attenuation	No	ON	Yangzhou University, China	HI serum titers and protective effectiveness in chicken	[120]
NDV structural proteins	Chimeric vaccine, HN and *F* genes alteration in an existing attenuated strain	No	IN, OC	Yangzhou University, China	Immunogenicity, HI serum titers and protective effectiveness in chicken	[122]
NDV structural proteins	Strain isolated from a wild duck	No	PO	Group of institutes, Russia	Immunogenicity and protective efficacy in chicken	[174]
Inactivated vaccine						
NDV structural proteins	Inactivation of a virulent strain	Montanide ISA 70	SC	Bangladesh Agricultural University, Bangladesh; Cornell University, Ithaca, USA	HI serum titers and protective effectiveness in chicken	[98]
NDV structural proteins	Inactivation of a virulent strain	PLGA	IM	ICAR-Indian Veterinary Research Institute, India	HI serum titers and protective effectiveness in chicken	[126]
NDV structural proteins	Inactivation of a virulent strain	Incomplete Freund’s adjuvant	SC	Bangladesh Agricultural University, Bangladesh	Immunogenicity and protective efficacy in chicken	[124]
HVT-vectored vaccine						
F protein	HVT-vectored vaccine expressing F protein	No	SC	FARVET S.A.C., Peru; The Pirbright Institute, UK	Immunogenicity and protective efficacy in chicken	[129]
F protein	HVT-vectored vaccine expressing F protein	No	SC	Yangzhou University, China;	Immunogenicity and protective efficacy in chicken, virus neutralizing activity of chicken serum in vitro	[130]
Adenovirus-vectored vaccine						
F protein	Adenovirus (Ad5) vectored vaccine expressing F protein	No	NI	US National Poultry Research Center, USA	HI serum titers and protective effectiveness in chicken	[131]
F protein	Adenovirus (Ad5) vectored vaccine expressing F protein	No	IM, SC, IN	McGill University, Canada; National Veterinary Institute, Ethiopia; Université Tunis El Manar, Tunisia	Protective efficacy in chicken	[135]
HN protein	Adenovirus (Ad5) vectored vaccine expressing HN and ChGM-CSF	ChGM-CSF	IM	Northwest A&F University, China	HI serum titers and protective effectiveness in chicken	[132]
F protein	Adenovirus (Ad5) vectored vaccine expressing F protein	No	IM	Zhaoqing Branch of Guangdong Laboratory of Lingnan Modern Agricultural Science and Technology and College of Veterinary Medicine, China	Immunogenicity and protective efficacy in chicken	[133]
Subunit recombinant vaccine						
F protein	Transgenic rice seeds	Montanide™ ISA 71 VG	IM	Northwest A&F University; Wuhan Healthgen Biotechnology Corp., China	Immunogenicity and protective efficacy in chicken	[146]
HN and F proteins	Tobacco hairy roots	No	PO	National Institute of Genetic Engineering and Biotechnology, Iran	Immunogenicity in mice	[148]
HN-F protein	Transgenic canola seeds	No	PO	National Institute of Genetic Engineering and Biotechnology, Iran	HI serum titers in chicken	[149]
HN and F proteins	Transgenic maize seeds	No	PO	University of the Punjab, Pakistan	Safety in rats	[175]
HN protein dimer	Expression in transgenic rice seeds	ISA 71VG	NI	Henan Agricultural University and Henan Academy of Agricultural Sciences China;	HI serum titers and protective effectiveness in chicken	[176]
F and HN proteins	Transient expression in maize	Chitosan	IP	Shiraz University, Higher Education Center of Eghlid and Shahid Bahonar University of Kerman, Iran	Immunogenicity in rabbits	[152]
HN protein	*E. coli*	No adjuvant and Freund’s adjuvant	SC	CEMB University of the Punjab, Pakistan	Immunogenicity in chicken	[155]
HN protein	*E. coli*	LTB and Freund’s adjuvant	IP	National Institute of Genetic Engineering and Biotechnology, Iran	Immunogenicity in mice	[160]
HN and F proteins	*E. coli*	LTB and Freund’s adjuvant	IP	National Institute of Genetic Engineering and Biotechnology, Iran	Immunogenicity in mice	[159]
F and HN proteins	*Lactococcus lactis* bacterial-like particles	No	IN	Jilin Agricultural University, China	Immunogenicity and protective efficacy in chicken	[177]
VLP vaccine						
M, HN and F protein	Expression in insect cells	Incomplete Freund’s adjuvant	NI	University of Tabriz, Iran	HI serum titers and protective effectiveness in chicken	[169]
HN and F protein	Transient expression in *N. benthamiana*	Emulsigen^®^-P adjuvantant	IM	University of Pretoria, Gauteng, Pretoria, South Africa	Immunogenicity in chicken, virus neutralizing activity of chicken serum in vitro	[170]
Bivalent VLPs: F protein NDV, IBV S1 and M protein	Expression in insect cells	No	NI	Sichuan University, China	Immunogenicity and protective efficacy in chicken	[172]
Peptide vaccine						
F and HN proteins	Chemical peptide synthesis	Alum	SC	Khyber Medical University, Pakistan	HI serum titers in mice and chicken	[164]
Live bacterial vaccine vector						
F protein	*S. typhimurium* χ11246	chIL-18	PO	Jilin Agricultural University, China	Immunogenicity and protective efficacy in chicken	[142]
HN protein	*L. casei*	No	PO	Jilin Agricultural University, China	HI serum titers and protective effectiveness in chicken	[141]
DNA-vaccine						
F protein	Plasmid-based expression	No	IM	Indian Veterinary Research Institute, India	Immunogenicity and protective efficacy in chicken, virus neutralizing activity of chicken serum in vitro	[178]
F protein	Plasmid-based expression	IL-12	IM or electroporation	South China Agricultural University, China	Immunogenicity and protective efficacy in chicken, virus neutralizing activity of chicken serum in vitro	[138]
F and HN proteins	Plasmid-based expression	IL-28b	IM or OC	Sokoine University of Agriculture, Tanzania; Virginia Tech, USA.	HI serum titers and protective effectiveness in chicken	[139]

Abbreviations: IP—intraperitoneal, IM—intramuscular, IN—intranasal, SC—subcutaneous, PO—per oral, ON—occulo-nasal, OC—ocular, IO—in ovo, NI—no information.

## Data Availability

No new data were created or analyzed in this study.
